# Mapping immunotherapy potential: spatial transcriptomics in the unraveling of tumor-immune microenvironments in head and neck squamous cell carcinoma

**DOI:** 10.3389/fimmu.2025.1568590

**Published:** 2025-04-08

**Authors:** Seo-Won Choi, Jeong Heon Kim, Jisu Hong, Minsu Kwon

**Affiliations:** Department of Otolaryngology-Head and Neck Surgery, Asan Medical Center, University of Ulsan College of Medicine, Seoul, Republic of Korea

**Keywords:** cancer immunotherapy, head and neck squamous cell carcinoma, immune checkpoint inhibitor, spatial transcriptomics, tumor-immune microenvironment

## Abstract

Head and neck squamous cell carcinoma (HNSCC) often exhibits poor response rates to immune checkpoint inhibitor (ICI) therapies, largely owing to the intricate composition and spatial organization of immune cells within the tumor-immune microenvironment (TIME). The diversity of immune cell populations, their spatial relationships, and dynamic interactions significantly influence the immunosuppressive nature of the TIME, thereby limiting the efficacy of immunotherapy. To address these challenges and enhance the therapeutic potential of ICIs in HNSCC, a comprehensive analysis of the TIME is essential. Spatial transcriptomics (ST), a cutting-edge technology, enables high-resolution mapping of gene expression within the spatial context of the tumor, providing critical insights into the functional roles and interactions of immune cells in the TIME. This review highlights the importance of ST in uncovering the complexities of the TIME in HNSCC and proposes strategies for leveraging these insights to develop more effective immunotherapeutic approaches. By integrating spatial and molecular information, this review aims to pave the way for personalized and precision-based treatments in HNSCC, ultimately improving patient outcomes.

## 1 Introduction

Head and neck squamous cell carcinoma (HNSCC) is a generic term that includes cancer occurring in the oral cavity, sinonasal cavity, pharynx, and larynx. It is the sixth most common cancer worldwide, and its incidence is steadily increasing, with a 30% expected increase by 2030 (1). The overall survival (OS) rate of patients with HNSCC has remained poor, varying between 30% to 70% (2). To face this challenge, the use of anti-cancer immunotherapy has emerged as a potential solution with the revolutionary development of therapeutics targeting components of the tumor-immune microenvironment (TIME). However, only 15-20% of patients with HNSCC benefit from immunotherapy with an immune checkpoint inhibitor (ICI) (3, 4). Furthermore, clinical prognosis is largely affected by multiple factors that reside within the tumor milieu, including the infection status of human papillomavirus (HPV) (1). The TIME of HNSCC based on HPV status exhibits a considerably heterogeneous immune cell composition (5). Collectively, it has become increasingly important to thoroughly investigate the TIME of HNSCC to improve the therapeutic efficacy of ICIs.

Several approaches have been used to explore the TIME. Early methods computationally de-convoluted gene expression from bulk RNA-seq to measure the proxy of immune cell proportion using several marker gene sets (6). However, owing to the varying source of RNAs captured by bulk RNA-seq, it is ambiguous whether the differences in gene expression imply uniform dysregulation of genes or changes in the cell population expressing the genes. Consequently, the low resolution of bulk RNA-seq led to the development of single-cell RNA-seq (scRNA-seq), which can specify the origin of the RNAs sequenced. The introduction of scRNA-seq to tumor samples revealed an astounding diversity of non-tumoral cells that infiltrate tumor tissue, shedding light on how each transcript is differentially regulated in each cell and, particularly, enabling quantitative and qualitative analysis of immune cells within the TIME (7, 8). In HNSCC, numerous studies have employed scRNA-seq to investigate tumor characteristics and predict patient prognosis. Especially, Puram et al. identified a partial epithelial-to-mesenchymal transition (p-EMT) program and proposed that the expression of p-EMT markers at the leading edge (LE) of primary tumors is closely linked to pro-tumoral changes in the TIME, affecting immune cells and stromal cells in a way that promotes tumor growth and immune evasion (9).

Despite these achievements, how each cell responds to its environment raises the question of how cells are spatially distributed and interact with various biological signals in the spatial context of the tumor. Consequently, the characterization of TIME and the prediction of patient prognosis quickly reached their limits without the spatial information of the cells comprising TIME. In a 2016 study, spatial transcriptomics (ST) emerged as a powerful way to fill this gap (10). ST can locate and visualize the profiles of RNA molecules in corresponding tissue in a two-dimensional manner simultaneously, thereby adding physical context to biological phenomena. Many ST techniques are based on either imaging, where single-molecule fluorescence *in situ* hybridization occurs, or on *in situ* capturing, in which mRNA molecules are bound to probes that coat “spots” on a slide (11). The use of ST has overcome many limitations of scRNA-seq by enabling in situ-profiling of gene expressions and greatly facilitated the transition of biological knowledge from scRNA-seq to clinical applications in oncology, as shown in pancreatic ductal adenocarcinoma (PDAC) and non-small cell lung cancer (12, 13). In addition, the transcriptional architectures in the tumor core (TC) and LE of HNSCC were different, and the differentially expressed gene signatures according to the tumor space could be a significant predictor of patient prognosis and response to ICIs (14, 15). Collectively, these studies highlight the complexity of the TIME in HNSCC and indicate the need for further investigation of the physical distribution of cells and their interaction in this environment.

In this review, we summarize the characteristics of the complex immune and stromal cells within the TIME of HNSCC and their impact on patient prognosis and therapeutic response. Furthermore, we discuss the indispensability and utility of ST in deciphering the intricate interactions among cells within the TIME, focusing on its potential to enhance the efficacy of anti-cancer immunotherapy. Lastly, we propose perspectives on combination therapies aimed at simultaneously targeting the therapy-resistant cells identified through ST within the TIME, to improve the outcomes of current cancer therapeutics against HNSCC.

## 2 Characteristics of the TIME of HNSCC: benefits of ST for deciphering it

### 2.1 Distinct immune cell composition and location

**Figure 1:**
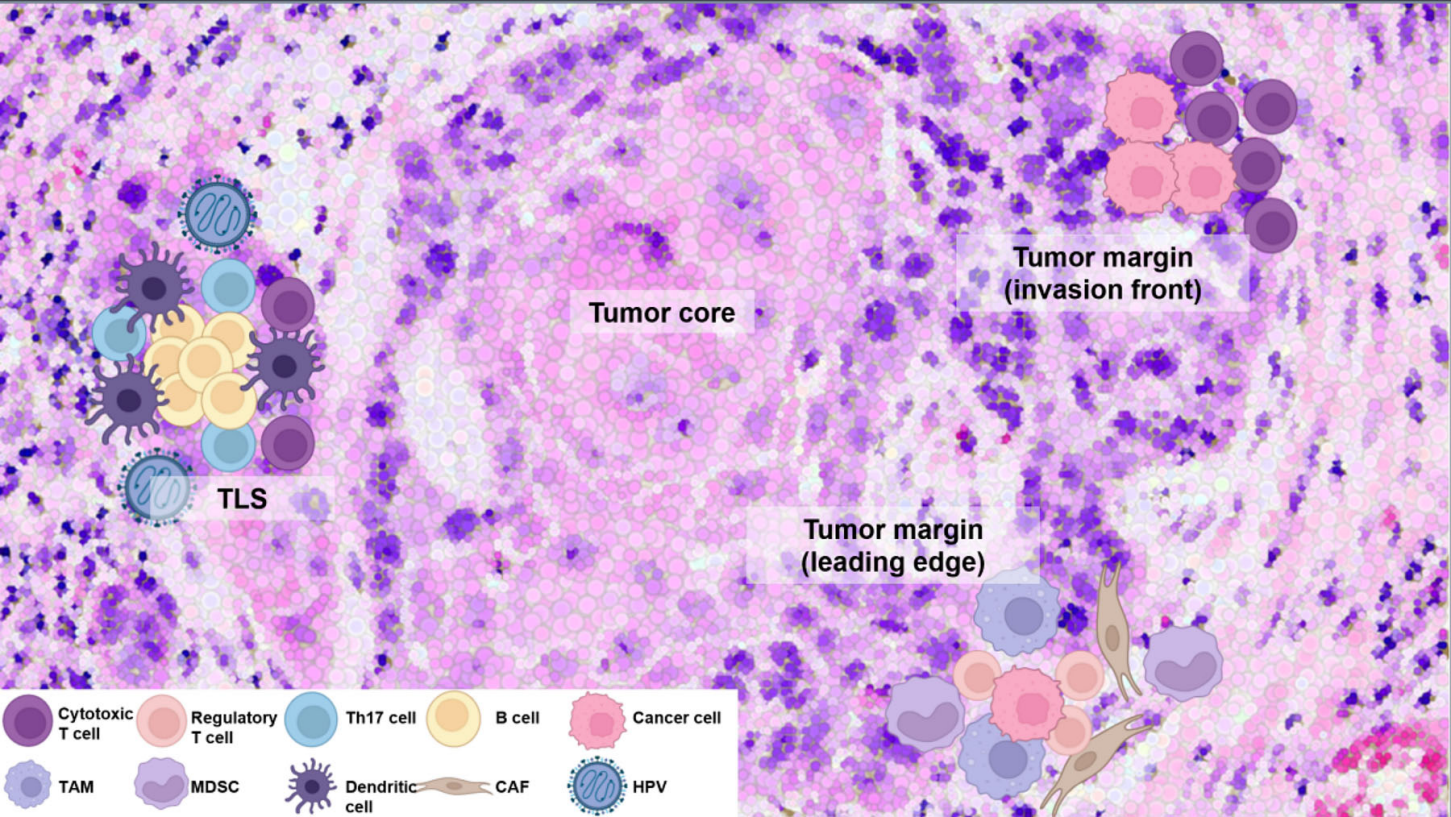
Distinct immune cell composition and spatial organization within the TIME of HNSCC. CD8^+^ cytotoxic T cells are enriched at invasion front of tumor, promoting anti-tumor immunity, whereas deeper tumor regions with active invasion activity (leading edge) are dominated by immunosuppressive regulatory T cells and M2-polarized tumor-associated macrophages (TAMs). Cancer-associated fibroblasts (CAFs) also play a pivotal role by remodeling the extracellular matrix, excluding CD8^+^ T cells, and fostering an immunosuppressive environment with interacting TAMs, further driving immune evasion and resistance. High-density B cells with IL-17-producing helper T (Th17) cells within the tertiary lymphoid structure (TLS) enhance anti-tumor activity, whereas TAMs and myeloid-derived suppressor cells (MDSCs) contribute to immune evasion. Human papillomavirus (HPV)-positive tumors show higher immune infiltration, particularly displaying the transcriptional signature of tumor-infiltrating B cells observed in germinal centers (GCs). This commonly observed phenomenon provides evidence of the association between HPV-positive tumors and the formation of TLS. These spatial and cellular variations underscore the need for tailored therapeutic approaches targeting specific immune and stromal cell interactions. This figure was created using tools provided by Biorender Illustration (https://app.biorender.com/illustration).

In HNSCC, the TIME is characterized by a diverse array of stromal cells and immune cells, including T cells, B cells, myeloid-derived suppressor cells (MDSCs), and tumor-associated macrophages (TAMs). Spatially, these cells are not randomly distributed, and their topological clustering can influence disease progression and response to therapies. For instance, CD8^+^ T cells, which exhibit anti-tumor activity, are often found at the invasion front of the tumor, the area adjacent to normal tissue. In contrast, the leading edge, which is the most advanced position in an area of invasion activity, is mainly dominated by immunosuppressive cells (9, 13). These cells, such as regulatory T cells (T_reg_s) and M2-polarized pro-tumoral TAMs, create immunosuppressive environments that inhibit the function of cytotoxic CD8^+^ T cells and promote tumor growth. Furthermore, areas with dense immune cell infiltration show condensed expression of immune checkpoint molecules such as programmed death receptor-1/programmed death ligand-1 (PD-1/PD-L1), which contribute to the exhaustion of T cells. This spatial heterogeneity of the immune landscape within the TIME plays a critical role in immune evasion, impacting the effectiveness of ICIs and other therapies (16, 17). Consequently, it is important to investigate the types of immune cells that determine the response to anti-cancer immunotherapy within the TIME of HNSCC and why their spatial characteristics are formed (**Figure 1**).

#### 2.1.1 T cells

In predicting responses to ICIs, tumor-infiltrating lymphocytes (TILs) have traditionally been categorized into immune-inflamed, immune-excluded, and immune-desert phenotypes based on their spatial distribution (18). However, beyond spatial localization, the functional characteristics of T cells, which form the majority of TILs, are critical determinants. In the TIME of HNSCC, five major T cell subsets have been identified: naïve-like, cytotoxic, pre-dysfunctional, terminally dysfunctional/exhausted, and cycling cells (19, 20). Among these, active cytotoxic T cells are indispensable for robust immunotherapy responses, irrespective of HPV status (1). Notably, all subsets except for naïve-like T cells are more abundant in the TIME compared to adjacent normal tissue, suggesting active T cell maturation within the TIME (20–23). Conversely, exhausted T cells (T_ex_s) and regulatory T cells (T_reg_s) contribute to an immune-suppressive or unresponsive environment, with a high representation of these subsets correlating with shorter overall survival (OS) in TCGA datasets of HNSCC (21, 23). T_ex_s display an ordered loss of effector functions along with the expression of inhibitory receptors such as PD-1, T-cell immunoglobulin and mucin-domain containing-3 (TIM-3), lymphocyte-activation gene-3 (LAG-3), cytotoxic T lymphocyte associated antigen-4 (CTLA-4), and T-cell immunoreceptor with immunoglobulin and ITIM domain (TIGIT) (1). On the other hand, high infiltration of follicular helper T (T_fh_) cells and interleukin (IL)-17−producing helper T (T_h17_) cells is strongly associated with improved overall and progression-free survival in patients with HNSCC. These cells enhance anti-tumor immunity by recruiting and activating B and T cells (24).

The development, differentiation, and functional regulation of these T cell subsets are critically governed by transcription factors. For example, Notch1 determines T cell lineage in the thymus and drives differentiation, whereas GATA Binding Protein 3 promotes CD4^+^ T cell development and T_h2_ differentiation through the production of IL-4, IL-5, and IL-13. T_cf1_ is essential for early T cell development and memory T cell formation. Retinoid orphan receptor gamma t drives T_h17_ differentiation, promoting inflammatory cytokines such as IL-17A and IL-22. Nuclear factor kappa B and nuclear factor of activated T-cells regulate cytokine production, T cell activation, and differentiation into Th subsets, whereas activating protein-1 promotes T cell survival and proliferation. Eomesodermin supports memory CD8^+^ T cell development and cytotoxicity. In mouse models, B-cell lymphoma/leukemia 11b maintains T cell identity by preventing alternative lineage differentiation. T-box expressed in T cells facilitates T_h1_ differentiation and interferon-gamma (IFN-γ) production. In addition, forkhead box P3 is pivotal for T_reg_ development and immune suppression to maintain self-tolerance and neurogenic locus notch homolog protein 1 determines T cell lineage in the thymus and drives differentiation. Thymocyte selection-associated high mobility group box protein (Tox) drives T cell exhaustion through the upregulation of inhibitory receptors in chronic infections and tumors (25). The expression of transcription factors, which dictate the functional states of T cells and regulate their differentiation, is determined by the spatial localization of T cells within the TIME and their interactions with surrounding cells (26). In summary, the interplay between the functional and spatial characteristics of T cells, as well as the transcription factors that govern their development and activity, plays a crucial role in shaping the immune landscape and determining responses to immunotherapy in HNSCC, emphasizing the need for a comprehensive evaluation of T cell dynamics within the TIME.

#### 2.1.2 B cells

B cells are highly versatile, performing functions such as antibody production, antigen presentation, initiation of tertiary lymphoid structure (TLS) formation, cytokine production, and direct cell lysis. ST have uncovered the complex roles of B cells in the TIME, indicating their dual contributions to cancer progression. In colorectal cancer, leucine-tRNA-synthase-2-expressing B cells have been identified as key players in promoting immune evasion, while in gastric cancer, detailed re-analysis of single cell and spatial data reveals a dynamic B cell landscape that engages in significant crosstalk with tumor cells, potentially impacting immunosuppressive TIME with subsequent poor clinical outcomes (27, 28). Within the TIME, B cells are predominantly found in TLSs and often co-localize with CD8^+^ T cells and follicular helper T (T_fh_) cells in these structures (1). Recent studies have demonstrated that in HNSCC caused by HPV infection, patients exhibit transcriptional signatures of germinal center (GC) tumor infiltrating B cells and spatial organization of immune cells consistent with TLS with GCs (**Figure 1**). And a high density of B cells, similar to active CD8^+^ T cells, is associated with favorable outcomes, including enriched GCs and longer progression-free survival as well as superior OS in both patients with HPV-positive and HPV-negative HNSCC (1, 29). Consequently, the spatial clustering of B cells within the TIME is a critical factor in TLS formation. To improve therapeutic efficacy in HNSCC, it is crucial to conduct studies that thoroughly explore and leverage the regulatory dynamics of B cells. However, research on TLS formation and B cell function in HNSCC remains limited, and the specific mechanisms and regulatory factors driving this process are poorly understood. Further investigation into the formation and regulation of TLS in HNSCC is essential, as it could lead to the development of novel therapeutic strategies targeting B cells to enhance anti-cancer immunity.

#### 2.1.3 TAMs and MDSCs

Macrophages are phagocytic cells with high plasticity, and their subsets are not identified *in vivo*. Their functions may vary according to phenotype rather than their origin, making their characterization even more challenging (30). Nevertheless, macrophages in TIME (TAMs) are mostly related to recurrent/metastatic (R/M) disease and poor patient outcomes (1). Within the HNSCC TIME, TAMs are one of the primary sources of PD-L1 and spatially co-localize with CD8^+^ T cells. In a study of 51 patients with HNSCC, TAMs, in addition to mast cells, were the only cell type with significant prognostic impact. Further analysis showed that the expression ratio of C-X-C motif chemokine ligand 9 (CXCL9) and secreted phosphoprotein 1 (SPP1) in TAMs provided a prognostic signature, independently of HPV status (15). Another group identified SPP1^+^ C-C motif chemokine ligand 18 (CCL18^+^) and SPP1^+^folate receptor 2 (FOLR2^+^) TAMs as a major platform of metastatic transcriptional programs and showed that they were correlated with poor survival outcomes of patients with HNSCC (31). An intriguing and important point is that these TAMs are characteristically located in the LE rather than the TC, and their characteristics enable them to influence the overall tumor properties through interactions with stromal cells distributed at the tumor margin. Taken together, spatially and transcriptionally characterized SPP1^+^ TAMs in the TIME of HNSCC seem to be one of the potential targets for successful immunotherapy.

MDSCs are a heterogeneous group of cells that are widely presented in the TIME. MDSCs are primarily classified into two major subtypes: polymorphonuclear (PMN)-MDSCs and monocytic (M)-MDSCs. PMN-MDSCs resemble neutrophils and are characterized by high levels of reactive oxygen species, arginase 1 (ARG1), and myeloperoxidase, primarily suppressing T cell proliferation. M-MDSCs resemble monocytes and are defined by high expression of inducible nitric oxide synthase and ARG1, producing nitric oxide to exert potent immunosuppressive effects and differentiate into TAMs (32). They mostly feature immunosuppressive functions, and some conventional drugs have been successful in depleting MDSCs and improving the efficacy of immunotherapy (33, 34). MDSCs promote various pro-tumoral effects via activation of T_reg_s and T_h2_ cells, inducing depletion of cysteine, production of arginine, and suppression of nature killer (NK) cell activity (35, 36). To date, few studies have specifically focused on the spatial transcriptomic characteristics of MDSCs in HNSCC. However, MDSCs play a critical role in therapeutic resistance and patient prognosis in HNSCC. This highlights the need for future research to elucidate their spatial dynamics and functional contributions within the TIME to better understand their impact on treatment outcomes.

#### 2.1.4 Cancer-associated fibroblasts

The most commonly found stromal cell types in TIME are lymphatic and vascular endothelial cells, mesenchymal stem cells, and CAFs (37). In multiple cancers, CAFs are typically observed to spatially colocalize with epithelial cells, contributing to the aggressive characteristics of tumors. In PDAC, CAFs colocalize with terminal ductal cell populations, TAMs, and dendritic cells (DCs) within spatially restricted enrichments (12). In bladder cancer, CAFs are localized in close proximity to cadherin 12^+^ epithelial cells and contribute to an immunosuppressive TIME (38). Additionally, in the deep invasive layer of diffuse-type gastric cancer, CCL2^+^ CAFs and endothelial cells are significantly enriched compared to the superficial layer, indicating the presence of immunosuppressive immune cell subtypes that may enhance tumor cell aggressiveness in these layers (39).A study with 20 patients with HNSCC deciphered the functional enrichment of CAF clusters, which included extracellular structure organization and collagen fibril organization. Interestingly, CAF signatures have a negative impact on the prognosis of patients with HPV-positive HNSCC but not with HPV-negative HNSCC (19). Another study performed by Obradovic et al. annotated 14 types of CAF populations with distinct changes in abundance after nivolumab treatment using bulk and single-cell samples of 32 patients with HNSCC. They also demonstrated that the CAF subpopulation can be predictive of treatment response and be associated with immunosuppression (40). Furthermore, a recent study revealed a subset of CAFs in HNSCC that is associated with the exclusion and dysfunction of CD8^+^ T cells. Using ST and scRNA-seq, the study identified that these CAFs express high levels of chemokines (CXCL9, CXCL10, and CXCL12) and Galectin-9, contributing to immune evasion and reduced T cell infiltration in tumors (41). Collectively, these findings underscore the multifaceted roles of CAFs and other stromal cells in HNSCC, including their contributions to tumor progression, immune evasion, and treatment resistance, highlighting their potential as both prognostic markers and therapeutic targets.

### 2.2 HPV association and its unique characteristics compared to the TIME of other cancers

HPV infection significantly impacts the TIME in HNSCC, leading to distinct differences compared to HPV-negative tumors (**Table 1**). HPV-positive tumors are typically infiltrated by a higher density of immune cells, including CD8^+^ T cells, CD4^+^ helper T (T_h_) cells, B cells, and NK cells, compared to HPV-negative tumors (42). This increased immune presence is associated with elevated expression of immune checkpoint molecules such as PD-1 and PD-L1, indicating an active immune response. Conversely, HPV-negative tumors often display a more immunosuppressive environment, characterized by higher infiltration of T_reg_s and MDSCs, which contribute to immune evasion (43). These distinct compositions of the TIME ultimately translate into differences in therapeutic responsiveness. That is, HPV-positive tumors typically exhibit a higher response rate to various treatments than HPV-negative tumors (44). For instance, high PD-1 expression on CD8^+^ TILs predicts responsiveness to anti–PD-1/PD-L1 therapies in patients with HPV-positive HNSCC, but not in HPV-negative cases (45). Conversely, HPV-negative tumors respond better to CDK4 inhibitors than HPV-positive tumors (46). In summary, the distinct immune profiles of HPV-positive and HPV-negative HNSCC significantly influence the composition of the TIME and result in different therapeutic responses.

**Table 1:** Differences in biological and clinical characteristics of HNSCC based on HPV status.

	HPV+ HNSCC	HPV- HNSCC
Cell composition	High TILs, CD8^+^ T cells, Th1 CD4^+^ T cells, B cells, NK cells; frequent TLS	High neutrophils, macrophages, monocytes, NK/T cells (often inactive)
Immune activation	Increased immune signaling pathways, cytotoxicity, T cell exhaustion markers	Immune suppression via TAMs, MDSCs, and higher tumor-associated antigens
Prognosis and therapy response	Better response to immunotherapy, improved prognosis	Resistance to immunotherapy, poorer prognosis
Mechanisms (key drivers)	Viral antigens (E6/E7), pro-inflammatory cytokines (IFN-γ, TNF-α)	Chronic inflammation (smoking/alcohol), self-antigen mutations, MDSC activity

HNSCC, head and neck squamous cell carcinoma; HPV, human papillomavirus; IFN, interferon; MDSC, myeloid-derived suppressor cell; TAM, tumor-associated macrophage; TIL, tumor infiltrating lymphocyte; TNF, tumor necrosis factor.

ST have provided deeper insights into these differences. In HPV-positive HNSCC, immune cells are more evenly distributed throughout the TIME, facilitating effective anti-tumor activity. In contrast, HPV-negative tumors exhibit compartmentalization of immune cells, with immunosuppressive cells predominantly located in the TC, creating localized regions that hinder immune cell infiltration and function (47). These spatial distributions are influenced by several factors. HPV-positive tumors express viral antigens that enhance immune cell recruitment and activation, leading to a more inflamed and responsive TIME. Additionally, the presence of TLS in HPV-positive tumors supports organized immune cell interactions and sustains anti-tumor immunity. In HPV-negative tumors, chronic exposure to carcinogens such as tobacco and alcohol induces an immunosuppressive milieu, promoting the accumulation of cells that inhibit effective immune responses (1). Understanding these spatial and cellular distinctions is crucial for developing targeted immunotherapies and improving prognostic assessments in patients with HNSCC. In summary, the distinct TIME characteristics in HPV-positive and HPV-negative HNSCC highlight the influence of HPV status on immune dynamics and therapeutic responses, underscoring the need for tailored treatment strategies.

### 2.3 Benefits of ST for understanding the TIME of HNSCC

#### 2.3.1 Co-localization and cell-to-cell interactions

As stated above, some cells tend to co-localize with specific cell types and interact with each other. For instance, PD-L1–positive TAMs co-localize with CD8^+^ T cells to communicate with each other (19). B cells were found to co-localize with both CD8^+^ T cells and T_fh_ cells in TLSs in patients with HNSCC, possibly indicating their close communication (29, 48–50). In samples with lymph node (LN) involvement, malignant cells, and T_reg_s colocalized more often, implying an unknown immunological impact of T_reg_s upon LNs (51). In addition, pEMT-high cells specifically localize to the LE of primary tumors and express ligands that are responsible for the pEMT transition of malignant cells (9, 52). Moreover, the distance between nerve and malignant cells was predictive of survival outcomes of perineural invasion-positive patients. Ligand-receptor analyses demonstrated that CAF-originated bone morphogenetic protein 4-activated bone morphogenetic protein receptor type 2 on cancer cells, promoting the pEMT program (9, 53). Other ligand-receptor interactions between CAFs and cancer cells, namely COL1A1-CD44 and LGALS7B-CXCL8, are responsible for HNSCC progression (54). Taken together, ST provides valuable insights into the spatial relationships and molecular interactions within the TIME of HNSCC, shedding light on mechanisms that drive tumor progression and immune modulation.

#### 2.3.2 Structural features: exploring the TLS

TLS is an ectopic structure mostly found at the invasive margin of tumors, composed of aggregated lymphocytes, mainly B and T cells (55). Despite being unencapsulated and transient, TLS shares many similar aspects with secondary lymphoid organs (SLO), such as LNs and the spleen (56). Although the formation of TLS is induced by a different reaction than that of SLO, the cytokines involved in both processes belong to the lymphotoxin and tumor necrosis factor families (56, 57). B and T cells form distinct zones inside TLS, resembling the organization in SLO (58). Interestingly, specialized macrophages (T cell membrane protein 4^+^FOLR2^+^) distinct from the triggering receptor expressed on myeloid cells 2^+^ macrophages infiltrating tumor nests are present in both the T cell and B cell zones of the TLS (59). The B cell zone often contains naïve B cells surrounding GCs, which are characterized by the infiltration of three subtypes of T_fh_ cells. High endothelial venules (HEVs), another common component of both TLS and SLO, serve as major gateways for the extravasation of circulating immune cells. HEVs may also facilitate the migration of immune cells from TLS to SLO, providing a potential explanation for the association of TLS with good clinical outcomes (55). TLS can be categorized into three developmental stages based on the composition and activation status of their immune cells. At the initial stage, TLS has minimal organization, with lymphoid aggregates and occasional DCs, but lacks follicular DCs (fDCs). The next stage, known as immature TLS or primary follicle-like TLS, contains more T and B cells with distinct zones, and a network of fDCs, but no GCs. Fully mature TLS, or secondary follicle-like TLS, exhibits active GCs, HEVs, and the ability to promote T and B cell activation, leading to TLS expansion through cell proliferation and recruitment (60, 61).

Although the presence of TLS is usually associated with a good prognosis in patients with HNSCC, their formation and maintenance require persistent tumor-dependent inflammation (56, 62–64). This presents a paradox, as chronic inflammation is generally pro-tumoral. The balance between the anti-tumoral actions of TLS and the pro-tumoral effects of the immune system may ultimately determine whether TLS results in a favorable outcome for patients with HNSCC (55). There are significant challenges in detecting TLS using different methodologies. For instance, TLS is easily identified through gene signatures in normal tissues but is challenging to detect using immunohistochemistry (65, 66). This highlights the importance of utilizing a wide range of markers to precisely detect TLS, with transcriptome profiling being a promising tool. Moreover, not only the presence of TLS but also the cellular patterns within TLS and their proximity to the tumor site significantly influence the response to ICIs (67–69). These findings emphasize the need to investigate both the biological and spatial aspects of TLS to fully understand their effects on TIME.

## 3 Immunotherapy in HNSCC: spatial clues in the TIME for overcoming therapeutic limitations

### 3.1 Current status of immunotherapy in HNSCC

#### 3.1.1 Types of ICIs and their efficacy

Currently, ICIs are approved only for patients with R/M HNSCC. Excluding patients who may be cured through salvage resection, re-irradiation, or metastasectomy, the remaining patients undergo first-line treatments that include ICIs (70, 71). The most common types of ICIs are anti–PD-1/PD-L1 inhibitors, which target the immune evasion mechanisms of cancer cells. Pembrolizumab, nivolumab, and durvalumab are currently approved and used as anti–PD-1/PD-L1 antibodies for treating patients with R/M HNSCC (4, 72, 73). Patients treated with pembrolizumab show durable overall response rates and non-inferior survival compared to a combination of platinum-based chemotherapy plus cetuximab (74). However, owing to the relatively lower response rate, pembrolizumab combined with chemotherapy is often used as a first-line treatment compared to ICI monotherapy. Anti–PD-1 antibodies can also be reconsidered as a second-line treatment for patients with platinum-refractory R/M HNSCC (75, 76).

Despite being widely utilized in clinical practice, ICIs face several significant challenges in the treatment of HNSCC. First, only approximately 18% of patients with HNSCC respond favorably to immunotherapy (3). Additionally, although ICIs are employed as a first-line treatment, the median OS reported in the KEYNOTE-048 study was limited to 12–14 months (74). Efforts to enhance the efficacy of ICIs, such as combining durvalumab with the anti–CTLA-4 antibody tremelimumab in clinical trials, failed to demonstrate an improvement in OS (73). Furthermore, though rare, some patients may experience hyperprogression (i.e., an accelerated progression of disease following ICI treatment) (77). Interestingly, even in such cases, subsequent chemotherapy has been shown to elicit durable responses (78). Furthermore, patients undergoing ICI therapy may also develop unexpected immune-related adverse events (irAEs), which necessitate treatment interruption and even lead to patient death (79). Collectively, these findings highlight the current limitations of ICIs-based immunotherapy in HNSCC, underscoring the need for continued efforts to overcome these challenges.

#### 3.1.2 Factors contributing to responsiveness to immunotherapies in HNSCC

In HNSCC, PD-L1 expression within tumor tissues is one of the most commonly used biomarkers for predicting responses to ICIs targeting the PD-1/PD-L1 axis. The combined positive score, which accounts for PD-L1 expression on tumor cells, lymphocytes, and macrophages relative to the total number of tumor cells, is currently used to estimate the likelihood of therapeutic efficacy (74, 80). Despite its widespread use, clinical studies have reported conflicting results regarding the reliability of PD-L1 as a biomarker. For instance, although some studies associate higher PD-L1 expression with improved OS in HNSCC, others suggest its predictive value is limited (81, 82). Consequently, emerging biomarkers beyond PD-L1 are being investigated in HNSCC. For example, the proportion of PD-1^+^ killer cell lectin like receptor G1^-^CD8^+^ T cells in peripheral blood strongly correlates with responses to neoadjuvant ICI therapy (83). Additionally, the accumulation of CD155^+^PD-L1^+^ MDSCs in HNSCC tumors has been linked to poor responses to ICIs. Notably, targeting the TIGIT-CD155 axis has shown promise in enhancing responses to PD-L1 blockade (84).

Tumor mutational burden (TMB) and neoantigen presentation are also critical factors influencing immune recognition in HNSCC (85). In the KEYNOTE-012 trial, patients with HNSCC and a high TMB (≥10 mutations per megabase) demonstrated significantly improved responsiveness to pembrolizumab (3). Neoantigens resulting from non-synonymous mutations can also trigger robust anti-tumor immune responses. However, only immunogenic mutations, not the overall mutational load, are strongly correlated with better outcomes following ICIs (86). HPV infection status is another established tumor-intrinsic biomarker in HNSCC, particularly in oropharyngeal squamous cell carcinoma. HPV-positive HNSCC tumors generally exhibit a more inflamed TIME, which would be theoretically associated with higher responsiveness to immunotherapy (87). However, landmark clinical trials conducted to date have reported no significant differences in response rates to ICIs based on HPV status, suggesting that HPV positivity alone may have limitations as a predictive marker for immunotherapy response (3, 4).

The gut and oral microbiomes have been increasingly recognized as potential determinants of therapeutic response to ICIs in HNSCC. These microbiomes influence anti-cancer immunity and therapeutic efficacy through the recruitment of small metabolites (88, 89). In a fecal microbiota transplantation study using mice, specific genera within the gut microbiome were identified as predictors of both immune-oncology therapy response and treatment-related toxicity (90). In both primary and metastatic HNSCC tissues, *Fusobacterium* species, prominent members of the oral microbiome, were found to be abundant, whereas beneficial *Streptococcus* species were notably reduced (91). Furthermore, in stage IV oral squamous cell carcinoma, an increased prevalence of *Fusobacterium periodonticum* and a decreased abundance of *Streptococcus mitis* and *Prevotella pasteri* were consistently observed (92). These findings suggest that specific microbial signatures in the gut and oral cavity could serve as biomarkers for predicting ICI treatment outcomes in patients with HNSCC.

According to a recent systematic review and meta-analysis by Kang et al., various biomarkers, including HPV positivity, PD-L1 expression, body mass index, albumin, Glasgow prognostic score, lactate dehydrogenase, neutrophil-lymphocyte ratio, and platelet-lymphocyte ratio, are associated with prognostic outcomes in patients with HNSCC treated with ICIs. However, few studies have focused on the TIME, MSI/MMR, hypoxia, and microbiome (93). These findings underscore the need for a more comprehensive and sophisticated approach that integrates tumor-intrinsic factors and TIME-related biomarkers to enhance patient selection and therapeutic outcomes in HNSCC.

#### 3.1.3 Causes of resistance to ICIs in HNSCC

Among patients with HNSCC, approximately 60% develop resistance to ICIs, with only 20–30% achieving long-term disease control. Resistance can arise through tumor-intrinsic or adaptive mechanisms, often driven by a combination of tumor genetics, environmental risk factors, and TIME characteristics (94). Tumor-mediated resistance mechanisms involve a variety of inhibitory pathways and immune evasion strategies. For example, the upregulation of LAG-3 impairs T cell proliferation and cytokine production, and similar immunosuppressive effects have been observed in pathways involving TIGIT, V-domain Ig suppressor of T cell activation, TIM-3, and CTLA-4. Specifically, TIM-3 and CTLA-4 overexpression has been implicated in T cell exhaustion, contributing to the immune escape of cancer cells. Furthermore, tumor cells can secrete indoleamine 2,3-dioxygenase (IDO1), which degrades L-arginine, a critical metabolite for the survival and proliferation of NK cells and T cells. Additionally, MDSCs and T_reg_s inhibit T cell function via nitric oxide signaling, whereas the Ikaros family zinc finger 1 and mitogen-activated protein kinase pathways reduce T cell recruitment, further depleting TILs (94).

Adaptive resistance often manifests through the upregulation of PD-L1 expression. For instance, activation of the phosphatidylinositol 3-kinase pathway by the yin yang 1 transcription factor enhances PD-L1 expression and promotes the production of decoy molecules that neutralize PD-L1 antibodies (95, 96). The NOD-like receptor pyrin domain-containing protein 3 inflammasome also contributes to increased PD-L1 expression (97). Independent of PD-L1, sex-determining region Y-box 2−mediated blockade of the type I interferon pathway can further impair immune activation, whereas mutations in transporter associated with antigen processing (TAP)-1, TAP-2, and human leukocyte antigen class I genes disrupt antigen presentation, preventing effective T cell priming (98). Numerous strategies to address resistance are under investigation. Manipulating the accumulation, function, and trafficking of MDSCs has shown partial efficacy in preclinical models. However, translating these findings into clinical practice has proven challenging owing to issues such as systemic toxicities and limited impacts on MDSC subsets (94, 99).

Stroma-mediated resistance, primarily driven by CAFs, creates a dense and fibrotic extracellular matrix (ECM) that serves as a physical barrier to immune cell infiltration. CAFs secrete factors such as transforming growth factor-beta (TGF-β) and vascular endothelial growth factor (VEGF), which promote vascular abnormalities and immune suppression. These vascular changes reduce immune cell trafficking to the tumor site and impair the efficacy of ICIs (41). Additionally, CAFs contribute to the polarization of macrophages toward an immunosuppressive M2 phenotype, further exacerbating resistance (100). Emerging therapies targeting stroma-mediated resistance are focused on modulating the CAF phenotype, normalizing the tumor vasculature, and enhancing immune cell infiltration. Although promising, these approaches require further optimization to overcome the heterogeneity of CAF populations and their diverse functions within the TIME (94).

### 3.2 Overcoming therapeutic limitations: Insights from spatial clues

ST techniques can illuminate the outcomes of cancer immunotherapies by deciphering components of the TIME, from which diverse cancer types have benefited. For instance, in glioblastoma, the population of sialic acid-binding Ig-like lectin 9^+^ TAMs observed in non-responders to immunotherapy could serve as a prognosticator of the outcomes (101). In non-small cell lung cancer, an enrichment of TAMs in pretreated patients was associated with resistance to immunotherapy, whereas responders exhibited an upregulated expression of CD25 in tumor regions (102, 103). SPP1^+^ TAMs colocalized with fibroblast activation protein (FAP)^+^ fibroblasts, and high expression of SPP1 or FAP correlated with diminished benefits from immunotherapy (104). Studies on clear cell renal cell cancer suggested that exhausted immune cells and the lack of PD-1, PD-L1, and CTLA-4 expressions could underline the non-responsiveness to immunotherapy (105).

**Figure 2:**
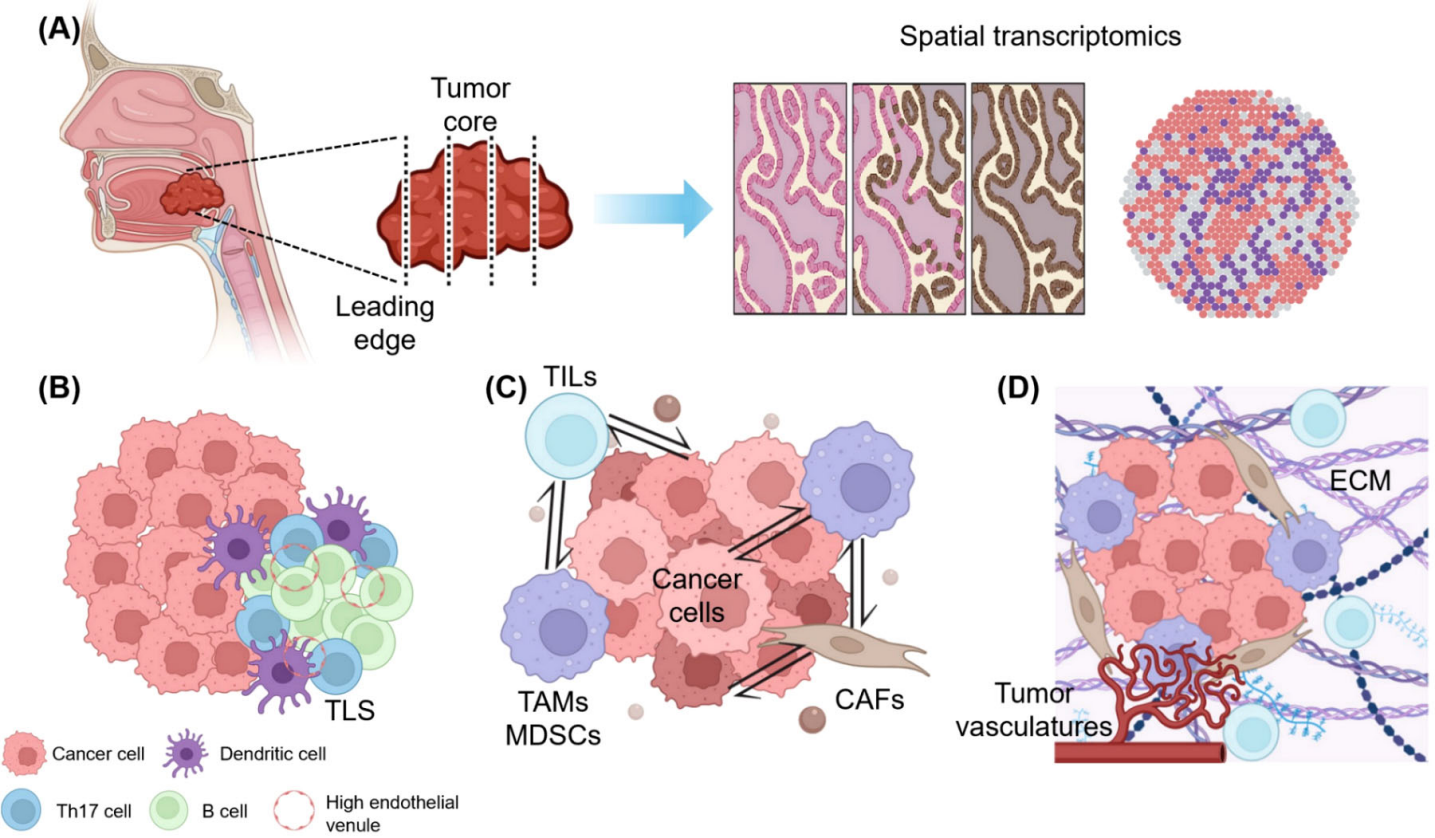
Overcoming therapeutic limitations in the immunotherapy of HNSCC: Insights from spatial clues. **(A)** Therapeutic targets from spatial transcriptomic data. Collected tumor samples are precisely analyzed considering their spatial location (whether from the tumor core or leading edge), and spatial transcriptomic information reveals key biomarkers and immune checkpoint molecules according to their spatially designated characteristics, aiding in patient selection and immunotherapy response prediction. **(B)** Precision medicine according to the location of tumor-infiltrating lymphocytes (TILs) and the presence of tertiary lymphoid structure (TLS). The distribution of TILs and TLS supports tailored immunotherapy strategies, with immune-excluded zones often indicating poor outcomes, whereas TLS-rich areas promote anti-tumor responses. **(C)** Spatially resolved cell-to-cell interactions and possible therapeutic strategies. Spatial mapping identifies immunosuppressive hubs formed by tumor-associated macrophages (TAMs), myeloid-derived suppressor cells (MDSCs), and cancer-associated fibroblasts (CAFs). Targeting TAM polarization and CAF-derived barriers enhances T cell infiltration and overcomes resistance. **(D)** Extracellular matrix (ECM) and vascular targeting treatment based on the topography of the tumor-immune microenvironment (TIME). ECM stiffness and abnormal angiogenesis create barriers to immune cell penetration. Targeting ECM components, such as collagen, and inhibiting vascular endothelial growth factor can normalize the TIME and synergize with immune checkpoint inhibitors for improved outcomes. This figure was created using tools provided by Biorender Illustration (https://app.biorender.com/illustration).

Given the highly heterogeneous nature of TIME in HNSCC, it is imperative to apply a multimodal treatment approach targeting its various components. Ongoing efforts are combining immunotherapy with other treatments to improve patient prognosis (**Table 2**). The combination of pembrolizumab with chemotherapy has been explored, though the 5-year OS rate remains around 20% (106). Additionally, clinical studies are investigating the effects of agonistic monoclonal antibodies targeting co-stimulatory molecules, such as OX40 (MEDI0562), CD137 (urelumab), and TLR8 (motolimod) (107). Furthermore, combinations of ICIs such as nivolumab and ipilimumab are being compared to standard care in several trials, including CheckMate 651 (NCT02741570) and NCT03700905 (108). Beyond ipilimumab, the anti-LAG3 antibody relatlimab is under evaluation for its effectiveness and safety, both alone and in combination with nivolumab (NCT01968109). Although these somewhat intuitive combinatorial approaches represent promising strides in targeting immune pathways, their efficacy often varies, underscoring the critical role of deciphering the TIME in shaping treatment outcomes. Therefore, as a more effective approach for cancer immunotherapy in HNSCC, we propose therapeutic strategies that consider the spatial characteristics and interactions of the components within TIME, as illustrated in **Figure 2**.

**Table 2:** Recent and ongoing ICI-based clinical trials in patients with HNSCC.

Trial ID	Target disease	Control	Experiment	Detailed results
NCT04247282	Previously untreated	M7824	M7824 with ETBX-011/ETBX-051+ ETBX-061/TriAd vaccine + Anktiva	86% (1-year RFS)
NCT03695510	Recurrent/metastatic	–	Afatinib + pembrolizumab	8.9 months (median OS)
NCT05383170	Advanced/metastatic	–	CyPep-1 + pembrolizumab	n.a.
NCT02764593	Locoregionally advanced	–	Nivolumab + cisplatin/Nivolumab + high-dose cisplatin/Nivolumab + cetuximab/Nivolumab + IMRT	n.a. n.a.
NCT03548467	Previously treated	VB10.NEO	VB10.NEO + bempegaldesleukin (NKTR-214)	n.a.
NCT03894891	Locoregionally advanced	–	Induction docetaxel + cisplatin + nivolumab (N) then Radioimmunotherapy (IMRT+N) then adjuvant N	12.9 months (median OS)
NCT04555837	Rb-deficient	–	Alisertib + pembrolizumab	16.8 months (median OS)
NCT03162224	Recurrent/metastatic HPV+	–	MEDI0457 + durvalumab	19.2 months (median OS)
NCT02578641	Advanced NPC	gemcitabine + carboplatin	Cytotoxic T cells+ gemcitabine + carboplatin	24.9 vs. 25 months (median OS)

HNSCC, head and neck squamous cell carcinoma; HPV, human papillomavirus; ICI, immune checkpoint inhibitor; IMRT, intensity modulated radiotherapy; n.a., not assessable; NPC, nasopharyngeal carcinoma; OS, overall survival; RFS, recurrence-free survival.

#### 3.2.1 Impact of spatial heterogeneity on immunotherapy in HNSCC

Spatial heterogeneity within the TIME significantly influences the efficacy of immunotherapy in HNSCC. Unlike other cancers, HNSCC is characterized by a highly dynamic and spatially variable immune landscape, where immune cells such as T cells, TAMs, and CAFs are unevenly distributed across the tumor (109). These spatial patterns create distinct immune niches, some promoting anti-tumor responses, whereas others foster immune evasion. For example, immune-excluded zones, where cytotoxic T cells are confined to stromal regions, are often associated with poor outcomes in anti-cancer immunotherapy (110). Moreover, traditional markers such as TILs and PD-L1 expression levels are gradually losing reliability as indicators for patient selection and response prediction. Instead, new spatially-informed biomarkers, such asT cell receptor productive clonality and TLS index, are emerging as promising indicators for predicting the efficacy of immunotherapy in patients with HNSCC (111). Moreover, the spatial distribution of stromal cells such as CAFs, and their impact on surrounding immune cells are also crucial factors determining the efficacy of immunotherapy against HNSCC (41). Additionally, the same authors recently reported that ST reveal the correlation between the TIME and overexpression of SLCO2A1 with tumor suppression in hypopharyngeal squamous cell carcinoma (112). Collectively, which patients with HNSCC are likely to respond to ICIs based on the unique spatial organization of their TIME can be better predicted by integrating spatial profiling techniques such as ST and multiplex immunohistochemistry.

#### 3.2.2 Targeting spatial interactions between immune and stromal cells to overcome resistance

The interplay between immune and stromal cells within the TIME is often dictated by spatial proximity and interactions. For instance, TAMs localized near CAF-enriched regions often form immunosuppressive hubs, which inhibit T cell activation and promote resistance to ICIs (113). Spatial mapping technologies enable a detailed visualization of these immune-suppressive networks, identifying key molecular mediators, such as transcription factors, cytokine gradients, micro-RNAs, and ECM components, that drive resistance. Armed with these insights, therapeutic strategies can be refined to target specific regions within the tumor. For example, depletion or reprogramming of TAMs from a pro-tumoral M2 phenotype to an anti-tumoral M1 phenotype can reduce the secretion of immunosuppressive cytokines such as IL-10 and TGF-β, thereby enhancing the activation and infiltration of effector T cells (114). Concurrently, altering CAF activity, such as inhibiting their secretion of ECM components and immunosuppressive factors such as CXCL12, can reduce physical and biochemical barriers that hinder immune cell penetration (115). These combined approaches promote the reactivation of T cells, thereby overcoming therapeutic resistance, including resistance to ICIs.

#### 3.2.3 Leveraging ST for combinational treatment strategies

ST has revolutionized our understanding of TIME, enabling the development of combinational treatment strategies tailored to specific spatial cellular contexts. For example, in hepatocellular carcinoma, Liu et al. integrated ST with scRNA-seq and multi-immunofluorescence to identify a tumor immune barrier (TIB) composed of SPP1^+^ macrophages and CAFs at the tumor boundary. This TIB was found to impede T cell infiltration, suggesting that targeting SPP1^+^ macrophages and CAFs could enhance the efficacy of ICIs by facilitating T cell access to the TC (116). In PDAC, Zhu et al. combined scRNA-seq with ST to reveal a TIME characterized by abundant immunosuppressive cells and dense stroma, which hinder effective immune responses. Their findings indicate that strategies aimed at reprogramming TAMs from an M2 to an M1 phenotype, alongside stromal remodeling agents, could potentiate the effects of ICIs in PDAC by mitigating immunosuppressive barriers and enhancing T cell infiltration (117). Furthermore, Cai’s study on breast cancer progression utilized single-cell and ST analyses to demonstrate that tumor cells express various co-inhibitory ligands interacting with immune cell receptors, fostering an immunosuppressive microenvironment. Spatial data confirmed the co-localization of tumor and immune cells expressing these ligand-receptor pairs, underscoring the potential of combinational therapies that block multiple co-inhibitory pathways to restore effective anti-tumor immunity (118).

ST has been used to develop combination therapies targeting various cells within the TIME of HNSCC. For example, Sadeghirad et al. utilized spatial profiling to identify immune checkpoint molecules and tumor necrosis factor receptor superfamily members as biomarkers of response to immunotherapy in HNSCC. Their findings suggest that targeting these molecules could enhance therapeutic efficacy (100). ST also revealed changes in the tumor and TIME in oral squamous cell carcinoma, providing insights into potential therapeutic targets (119). In addition, Li et al. reported that spatial analysis in HNSCC identified interferon-induced MHC-IhiGal9+ CAFs, which create a trap for CD8^+^ T cells and lead to cancer immune evasion, could be promising targets for more effective immunotherapy for HNSCC (41). These studies exemplify how ST data can inform combinational treatment strategies tailored to the unique spatial and cellular architecture of the TIME, thereby enhancing therapeutic efficacy across various cancer types including HNSCC.

#### 3.2.4 Modulating the ECM and angiogenesis to enhance immunotherapy outcomes

ST provides in-depth insights into the tissue architecture within the TIME. The physical barriers created by the ECM are among the key factors influencing the efficacy of therapies including ICIs. These ECM elements often create physical barriers that impede immune cell infiltration, thereby reducing the efficacy of immunotherapies. By identifying these structural impediments, targeted combination therapies can be developed to enhance treatment outcomes. For instance, the degradation of hyaluronic acid using hyaluronidase reduces ECM density, facilitating better penetration of immune cells into the tumor. This approach can be combined with ICIs to improve their effectiveness (120). Similarly, targeting collagen within the ECM can modulate the TIME to be more receptive to immunotherapy. ECM stiffness, primarily owing to collagen deposition, contributes to immune evasion. By targeting ECM stiffness and mechanotransducers, the TIME can be reprogrammed to enhance the efficacy of cancer therapies (121). Additionally, abnormal angiogenesis within tumors leads to the formation of dysfunctional blood vessels, creating hypoxic conditions that further suppress immune responses. VEGF plays a pivotal role in this process. Inhibiting VEGF can normalize tumor vasculature, improving immune cell infiltration and enhancing the effectiveness of ICIs. Clinical studies have demonstrated that combining VEGF inhibitors with ICIs results in synergistic anti-tumor effects across various cancers (122, 123). Recent studies regarding VEGF inhibitors on HNSCC treatment highlighted the interplay between the VEGF pathway and the TIME of HNSCC, demonstrating that combining VEGF inhibitors such as ramucirumab with ICIs such as pembrolizumab can modulate the TIME, enhance immune infiltration, and improve therapeutic efficacy in recurrent or metastatic HNSCC (124, 125). In summary, ST enables the precise identification of ECM components and vascular abnormalities within the TIME. This information is crucial for designing combination therapies that target these structural barriers, thereby enhancing the efficacy of immunotherapies. By integrating ECM-modifying agents or angiogenesis inhibitors with ICIs, it is possible to remodel the TIME to support robust anti-tumor immune responses against HNSCC.

## 4 Conclusions and perspectives

Understanding the spatial heterogeneity of the TIME represents a paradigm shift in the development of cancer treatment strategies. Spatial mapping provides crucial insights into the intricate interplay between immune and stromal cells, offering a deeper understanding of the immunosuppressive mechanisms within the TIME. Despite these advancements, few studies have utilized ST in HNSCC. The complex and heterogeneous nature of the HNSCC TIME poses significant challenges to effective treatment, as the spatial arrangement of immune and stromal components often contributes to therapeutic resistance. Decoding this intricate TIME architecture is essential for improving treatment outcomes in patients with HNSCC. ST, coupled with other spatially resolved technologies, enables the identification of localized immunosuppressive niches, such as immune-excluded regions or hypoxic zones, which are associated with resistance to ICIs. By integrating this spatial information, precision medicine approaches can be developed to tailor therapies to the unique TIME of each patient. Future advancements in spatial resolution technologies will further enable the discovery of novel biomarkers and therapeutic targets within the HNSCC TIME. These innovations will not only support the design of personalized treatment regimens but also address the unmet need to overcome therapeutic resistance in HNSCC. As ST research expands, the ability to decode the complex TIME of HNSCC will become indispensable for achieving better outcomes for patients facing this challenging cancer.
